# Strategies to improve the implementation of intensive lifestyle interventions for obesity

**DOI:** 10.3389/fpubh.2023.1202545

**Published:** 2023-07-25

**Authors:** Emily Benjamin Finn, Christine Whang, Peter Houlin Hong, Sergio A. Costa, Emily A. Callahan, Terry T. -K. Huang

**Affiliations:** ^1^Center for Systems and Community Design and NYU-CUNY Prevention Research Center, Graduate School of Public Health and Health Policy, City University of New York, New York, NY, United States; ^2^EAC Health and Nutrition, LLC, Leesburg, VA, United States

**Keywords:** obesity, lifestyle intervention, behavior change, human-centered design, implementation science

## 1. Introduction

Obesity is one of the most consequential diseases in the United States given its prevalence, long-term consequences, and high costs. More than 40% of U.S. adults and more than one-fifth (21.5%) of children aged 2–19 years have obesity, with worsening trends of inequities by race, ethnicity, socio-economic status, and geography (e.g., rural vs. urban) (1–3). People with obesity are at higher risks of multiple comorbidities, including cardiovascular disease, Type 2 diabetes, fatty liver disease, some forms of cancer, and depression (4, 5). The social consequences of obesity include discrimination, stigma and adverse impacts on education and employment (6, 7). The U.S. annual economic burden of obesity, including direct (health-related) and indirect (lost productivity) costs, has been estimated to be as high as $1.72 trillion (8).

First-line clinical management of obesity typically consists of multi-component, intensive lifestyle interventions (ILIs) that combine nutrition, physical activity, and behavior change support (9). Based on recommendations from authoritative bodies such as the U.S Community Preventive Services Task Force and the American Academy of Pediatrics, these interventions are often based on social cognitive models, including health education, goal setting, and social support (e.g., coaching) and can include motivational incentives such as rewards and behavioral feedback mechanisms (10, 11). Although several ILIs have demonstrated efficacy in clinical trials, their real-world impact, especially over the long-term, has been more limited; post-ILI weight regain is not uncommon. One systematic review demonstrated that some patients begin to experience weight regain around 36 weeks post-intervention, and that many participants regain all lost weight by 48 weeks (12–17). The persistent increase in rates of obesity suggests that we need to critically examine and directly address challenges in ILI design and delivery.

The goal of this opinion paper is to identify shortcomings in current ILIs, or “pain points,” experienced by patients—particularly those in underserved or minoritized communities—affected by obesity in the context of ILI implementation, that have not been adequately addressed to date. This paper is not meant to be an exhaustive discussion but reflects insights from the authors' research and reflections on the literature. The limitations are organized into three domains: (1) implementation context, (2) intervention components, targets, and sequencing, and (3) delivery strategies.

## 2. Implementation context

### 2.1. Mismatch between intervention and community readiness

Community readiness is defined by a community's preparedness to undertake change, which is influenced by its perceived importance of the health issue in question as well as its capacities, resources, and political climate (18). Low community readiness diminishes an intervention's impact potential. The Community Readiness Model (CRM), developed to guide the creation of community-level behavior change interventions, has been applied to a wide range of public health topics, including alcohol abuse, cardiovascular disease, and children's social-emotional development (19–22). A community's readiness varies by issue; for obesity, community readiness is often low or middling, in part because people may discount negative consquences of obesity that will accrue in the distant future (23–25). In this context, ILIs that target weight-related behaviors (e.g., diet or physical activity) may fail if they are not aligned with the community's highest priorities. Moving a community through stages of readiness can itself be an intervention outcome (18, 26). For example, baseline CRM findings were used to tailor a youth advocacy intervention to increase the community readiness for obesity prevention (27, 28).

### 2.2. Overlooking the built and social environment in a community

One premise of community readiness is that some communities may be unable to focus on obesity and its longer-term consequences until more immediate threats to safety and health (e.g., housing insecurity, crime, etc.) are addressed. Research has shown the importance of the role of built (e.g., distribution of food outlets) and social (e.g., perceived safety) environments in obesity. Few interventions, however, have incorporated environmental change as a critical component of ILIs (29–31). Dietz (32) cites “social conditions”, including safety, participant trauma, and housing insecurity, as a reason that seemingly promising ILIs have failed to improve body mass index (BMI) in low-income communities. For example, programs that focus on physical activity but do not account for neighborhood safety, park accessibility, quality of sidewalks or other environmental factors are unlikely to succeed (33). Environmental change often necessitates local policy intervention and cross-sectoral partnerships, which should be incorporated to a greater extent in the next generation of ILIs (34). The use of community health workers (CHWs) or patient navigators may also help patients address social determinants of health by linking patients to clinical and social services (35).

### 2.3. Lack of attention to cultural nuance

Evidence-based ILIs are often assumed to be equally effective across different populations. In reality, systems, cultural phenomena, and lived experiences shape unique pathways to obesity, which warrants culturally responsive design for evidence-based interventions (36). For example, among Chinese-American residents in Manhattan's Chinatown, grandparents are key caretakers and strongly influence young children's dietary intake. Many Chinese grandparents believe that chubby babies are healthy babies, partly due to historical experiences of food insecurity; this perception can lead to over-feeding. It is also common for newly immigrated parents, who often work multiple jobs, to send their newborns back to China to be raised by grandparents until children reach school age. Given these cultural nuances, ILIs developed in predominantly white populations may not be as effective with newly immigrant Chinese American families if interventions fail to engage grandparents in the U.S. or China (37). The use of culturally and linguistically concordant CHWs may help to bridge this cultural divide (35). In addition, the need for ILI adaptations is increasingly recognized in implementation science; however, how to optimally effect these adaptations is the next frontier of research (38).

## 3. Intervention components, targets, and sequencing

### 3.1. Discounting different individual stages of change for different behaviors

As with community readiness, many interventions fail to account for differences in participants' stages of change for various behaviors. The Transtheoretical Model, which maps individual behavior change progressing through six stages of change (SOC) (39), has been widely applied to obesity prevention and weight management efforts (40–43). However, individual SOC is behavior-specific: one may eat fruits and vegetables daily (action or maintenance stage) but not exercise (pre-contemplation or contemplation stage). As such, ILIs, which are designed to simultaneously address multiple aspects of obesity (e.g., physical activity, nutrition, sleep, etc.), must consider participant SOC relative to each behavior. Training for interventionists should include how to recognize participant SOC for each behavior and to tailor the intervention to each SOC.

### 3.2. Overlooking participants' mental health, cognitive load, and executive functioning

Mental health influences individual engagement with and response to ILIs. Weight stigma, low self-esteem, and mental health disorders (e.g., depression, anxiety) can all impede motivation, which is critical to individual movement through the SOC (44–46). Chronic stress interferes with self-regulation by decreasing physical activity, impairing sleep, and facilitating unhealthy eating (47, 48). Compared to other factors, poor mental health is a stronger predictor of attrition in obesity interventions, and mental health and weight loss have been strongly correlated, in some cases up to 12 months—and likely longer—after beginning ILIs (49, 50).

Cognitive load refers to the amount of information the brain can simultaneously process. It is not only negatively associated with mental health, but also limits ILI reach and uptake as people face competing demands that can become barriers to participation in obesity interventions (51, 52). Cognitive overload can result in decreased cognitive functioning. As such, participants who are cognitively overloaded before an ILI is introduced may be less likely to process the intervention effectively (53, 54). Teaching strategies developed specifically to avoid contributing to cognitive overload can be applied during the intervention design phase (55).

ILIs will be most effective when they address mental health and minimize cognitive load; more research is needed on this topic. The few pilot studies that have assessed the extent to which supporting participant stress management affected weight loss had small samples, short durations, and low generalizability (56–58). Studies with more robust statistical power as well as repeated longer-term measures of stress and mental health, cognitive functioning, health behaviors, and body composition would inform more effective ILI development. Qualitative studies are also needed to better contextualize the life experiences of intervention participants, so that desired behaviors such as healthy eating and physical activity can be considered as part of other life demands and stresses rather than be treated in isolation.

### 3.3. Knowledge gaps around the optimal sequencing of intervention components

Despite significant research around prevention and management of obesity, the optimal ways to combine ILI components remain under-explored (59). Most weight interventions target only one or two of the many factors associated with obesity; diet and physical activity are the most common but the importance of other behaviors, such as sleep, is increasingly recognized (60). The complexity of obesity demands broad-based interventions, but more research is needed on how to optimize the combination and sequencing of multiple intervention components, taking into account that such optimization may vary across populations. Several methodologies are available to explore optimal sequencing. Multiphase optimization strategy trials enable rigorous exploration of sequencing intervention components for obesity (61–64). The Fogg Behavior Model (FBM), which suggests that a behavior happens when motivation, ability, and prompt occur simultaneously, has been effectively applied to interventions for multiple health challenges. The FBM may enable obesity interventionists to more effectively support desired behavior changes, including by considering the specific sequencing of small behavioral steps before or after a current habit, leading to greater uptake of the target behavior (65–69). Adaptive interventions can be tailored to individual participant preferences and needs, and can be used to address early non-responders by modifying intervention intensity or form (70–72). Lastly, the use of simulation models in systems science has been applied to support optimization of intervention sequencing across several public health areas and could be similarly applied to obesity (73–75).

## 4. Delivery strategies

Interventionists must consider several aspects of ILI delivery. One is whether intervention materials are actively distributed (“pushed”) or must be actively sought by the participant (“pulled”). While comparisons of “push” vs. “pull” modalities have been studied, further research is needed to better understand when and where to use each type (76). For instance, one study found that patients and clinicians had opposite preferences, with patients preferring “pulling” information on resources, and clinicians preferring “pushing” (77). However, pull and push strategies may also be used together, for example, using an opt-out approach for intervention enrollment to maximize reach while allowing patients to customize some of the content or its presentation in the intervention.

Another aspect is whether interventions are delivered virtually or in-person. The pivot of in-person ILIs to virtual delivery at the onset of the COVID-19 pandemic provided a natural experiment to compare these modes, and early evidence sugguests this virtual transition may work for some programs but perhaps not others if staff and/or participants lack the technological self-efficacy to engage digitally, or if staff do not have the bandwidth to gain the required technological know-how (78). Currently, the U.S. Community Preventive Services Task Force recommends digital health interventions to improve diet and physical activity in workplace, higher education and community settings (10). However, with the exception of adolescents for whom this recommendation is made specifically to reduce overweight or obesity, the other companion recommendations are made to improve lifestyle behaviors only.

The field of lifestyle medicine has put forth successful practices related to the delivery of ILIs for obesity in clinical settings, such as shared medical appointments (79). In addition, the American College of Lifestyle Medicine released a statement in April 2023 stating that lifestyle interventions to treat obesity are often inadequately dosed for success (as it relates to the six pillars of lifestyle medicine used to treat chronic conditions, including obesity), indicating that delivery strategies must carefully consider dose (80).

Last but not least, interventionists must consider new and creative ways to meet intended participants where they are, physically, emotionally, and socially. Manga comics, exergaming (i.e., video games with interactive physical activity), and weight management podcasts have been used to engage various groups with promising results (81–83). Similar creativity for in-person interventions could facilitate embedding interventions into participants' day-to-day life. For instance, nutrition components of ILIs could be offered through parent and child engagement during grocery trips. Exercise components for adults could rely on brief “exercise snacks” instead of longer bouts of physical activity (84). The current evidence base for such strategies is limited.

## 5. Discussion

This opinion paper is focused on critical considerations for improving implementation and impact of obesity-related ILIs. The points raised have been understudied in the obesity field and warrant further research and development.

As the field moves toward the next generation of ILIs, further attention to the metrics used to gauge ILI success is important (85, 86). For example, given intra-individual variations in SOC in the context of multi-component interventions, a participant could become more physically active without making dietary changes while another could experience the reverse—but both individuals may experience only minor changes in BMI, although the intervention has improved their health to some degree. The selection of intervention success metric(s) can be influenced by the target population, goal(s) of the intervention recipients, intervention type, and intervention duration, among other factors.

As obesity interventions scale, the expected magnitude of behavior or health changes needs to be considred in line with the reach of ILIs. For instance, many digital interventions (e.g., web- or app-based) have had low or no statistically significant effect on participant weight despite high acceptability and feasibility (82, 87–90). Considering near-universal cell phone ownership, these types of interventions may offer wider benefit on a population level than the literature acknowledges, especially when offered in addition to standard clinical care rather than a stand-alone intervention (82, 87, 91–93).

The rise of implementation science (IS) has informed early efforts to improve the adoption, implementation, and sustainment of evidence-based interventions (94). IS offers a variety of frameworks and theories that can help design better interventions, improve outcomes or guide evaluations (95). Most IS frameworks are top-down and deterministic rather than bottom-up and human-centered. Although some recent advances have been made to address patient or community engagement in intervention implementation (96, 97), “how to” solve for patient pain points is still a major inquiry. Note that patient pain points may be affected by other concomitant chronic diseases or competing life priorities, which may influence the motivation to engage in obesity interventions. Human-centered design (HCD) can help bridge this gap in establishing program-context fit. HCD is a process for innovation by which design of a product or system incorporates end-user needs, preferences, and usage to develop a product that solves the user's “pain point” (98). HCD has slowly entered public health practice as a way to solve implementation challenges identified by IS frameworks. For example, Haines et al. (99) employed a three-step process of “usability testing,” “ethnographic contextual inquiry,” and “iterative prototyping” to ensure that their intervention, including its delivery methods, was best designed to fit the implementation context. Though more research is needed, HCD may allow for more innovative, strategic, and contextually tailored intervention designs, which may increase participant adoption and maintenance of health behaviors.

For obesity interventions to be successful in producing long-term changes in greater numbers of people, more effective, customized, and engaging interventions are needed. We hope this opinion will help move the field toward this end.

## Author contributions

TH conceived of this work, which was drafted by EF, PH, and CW, and substantially revised by TH, PH, CW, SC, and EC. All authors read and approved the submitted version of the manuscript.

## References

[1] Centers for Disease Control Prevention. Overweight and Obesity/Data and Statistics. (2022). Available online at: https://www.cdc.gov/obesity/data/index.html (accessed September 9, 2022).

[2] Fryar CD Carroll MD Afful J. Prevalence of Overweight, Obesity, Severe Obesity Among Children Adolescents Aged 2–19 Years: United States, 1963–1965 Through 2017–2018. Hyattsville, MD: National Center for Health Statistics (2020). Available online at: https://www.cdc.gov/nchs/data/hestat/obesity-child-17-18/obesity-child.htm

[3] Hu K Staiano AE. Trends in obesity prevalence among children and adolescents aged 2 to 19 years in the US from 2011 to 2020. JAMA Pediatr. (2022) 176:1037–9. 10.1001/jamapediatrics.2022.2052 35877133 PMC9315946

[4] Andolfi C Fisichella PM. Epidemiology of obesity and associated comorbidities. J Laparoendoscopic Adv Surg Techniq A. (2018) 28:919–24. 10.1089/lap.2018.0380 30010474

[5] Apovian CM. Obesity: definition, comorbidities, causes, and burden. Am J Manag Care. (2016) 22:s176–85. Available online at: https://www.ajmc.com/view/obesity-definition-comorbidities-causes-burden 27356115

[6] Centers for Disease Control Prevention. Obesity Basics / Consequences of Obesity. (2022). Available online at: https://www.cdc.gov/obesity/basics/consequences.html (accessed September 9, 2022).

[7] Puhl RM Heuer CA Brownell KD. Stigma and social consequences of obesity. In:Kopelman PG Caterson ID Dietz WH, editors. Clinical Obesity in Adults and Children. Hoboken, NJ: Wiley Blackwell (2010). p. 25–40.

[8] Waters H Graf M. America's Obesity Crisis: The Health Economic Costs of Excess Weight. Washington, DC: Milken Institute (2018). Available online at: https://milkeninstitute.org/sites/default/files/reports-pdf/Mi-Americas-Obesity-Crisis-WEB_2.pdf

[9] Kahan S. Overweight and obesity management strategies. Am J Manag Care. (2016) 22:s186–96. Available online at: https://www.ajmc.com/view/overweight-and-obesity-management-strategies 27356116

[10] Obesity: The Community Guide. Available online at: https://www.thecommunityguide.org/topics/obesity.html#cc-widget-45ec (accessed June 28, 2023).

[11] Hampl SE Hassink SG Skinner AC Armstrong SC Barlow SE Bolling CF. Clinical practice guideline for the evaluation and treatment of children and adolescents with obesity. Pediatrics. (2023) 151:e2022060640. 10.1542/peds.2022-060640 36622115

[12] Savoye M Shaw M Dziura J Tamborlane WV Rose P Guandalini C. Effects of a weight management program on body composition and metabolic parameters in overweight children: a randomized controlled trial. Jama. (2007) 297:2697–704. 10.1001/jama.297.24.2697 17595270

[13] Sacher PM Kolotourou M Chadwick PM Cole TJ Lawson MS Lucas A. Randomized controlled trial of the MEND program: a family-based community intervention for childhood obesity. Obesity. (2010) 18 (Suppl. 1):S62–8. 10.1038/oby.2009.433 20107463

[14] Curioni CC Lourenço PM. Long-term weight loss after diet and exercise: a systematic review. Int J Obes. (2005) 29:1168–74. 10.1038/sj.ijo.0803015 15925949

[15] Barte JC ter Bogt NC Bogers RP Teixeira PJ Blissmer B Mori TA. Maintenance of weight loss after lifestyle interventions for overweight and obesity, a systematic review. Obes Rev. (2010) 11:899–906. 10.1111/j.1467-789X.2010.00740.x 20345430

[16] Machado AM Guimarães NS Bocardi VB da Silva TPR Carmo ASD Menezes MC. Understanding weight regain after a nutritional weight loss intervention: Systematic review and meta-analysis. Clin Nutr ESPEN. (2022) 49:138–53. 10.1016/j.clnesp.2022.03.020 35623805

[17] Hall KD Kahan S. Maintenance of lost weight and long-term management of obesity. Med Clin North Am. (2018) 102:183–97. 10.1016/j.mcna.2017.08.012 29156185 PMC5764193

[18] Plested BA Thurman P Edwards R Oetting E Jumper-Thurman P. Community readiness: a tool for effective community-based prevention. Prev Res. (1998) 5:5–7.

[19] Paltzer J Black P Moberg DP. Evaluating community readiness to implement environmental and policy-based alcohol abuse prevention strategies in Wisconsin. J Alcohol Drug Educ. (2013) 57:27–50. Available online at: https://www.ncbi.nlm.nih.gov/pmc/articles/PMC4204645/pdf/nihms521333.pdf 25346555 PMC4204645

[20] Peercy M Gray J Thurman PJ Plested B. Community readiness: an effective model for tribal engagement in prevention of cardiovascular disease. Fam Community Health. (2010) 33:238–47. 10.1097/FCH.0b013e3181e4bca9 20531104

[21] Borrayo EA. Using a community readiness model to help overcome breast health disparities among U.S Latinas. Subst Use Misuse. (2007) 42:603–19. 10.1080/10826080701202205 17558953

[22] Islam S Small N Bryant M Bridges S Hancock N Dickerson J. Assessing community readiness for early intervention programmes to promote social and emotional health in children. Health Expect. (2019) 22:575–84. 10.1111/hex.12887 30972905 PMC6543141

[23] Pradeilles R Rousham EK Norris SA Kesten JM Griffiths PL. Community readiness for adolescents' overweight and obesity prevention is low in urban South Africa: a case study. BMC Public Health. (2016) 16:763. 10.1186/s12889-016-3451-9 27515802 PMC4982405

[24] Findholt N. Application of the community readiness model for childhood obesity prevention. Public Health Nurs. (2007) 24:565–70. 10.1111/j.1525-1446.2007.00669.x 17973734

[25] Schröder M Schnabel M Hassel H Babitsch B. Application of the community readiness model for childhood obesity prevention: a scoping review. Health Promot Int. (2022) 37:1–10. 10.1093/heapro/daac120 36047636

[26] Edwards RW Jumper-Thurman P Plested BA Oetting ER Swanson L. Community readiness: research to practice. J Commun Psychol. (2000) 28:291–307. 10.1002/(SICI)1520-6629(200005)28:3<291::AID-JCOP5>3.0.CO;2-9

[27] Frerichs L Brittin J Robbins R Steenson S Stewart C Fisher C. SaludABLEOmaha: improving readiness to address obesity through healthy lifestyle in a Midwestern Latino community, 2011-2013. Prevent Chronic Dis. (2015) 12:E20. 10.5888/pcd12.140328 25674679 PMC4329951

[28] Frerichs L Brittin J Stewart C Robbins R Riggs C Mayberger S. SaludableOmaha: development of a youth advocacy initiative to increase community readiness for obesity prevention, 2011-2012. Prevent Chron Dis. (2012) 9:E173. 10.5888/pcd9.120095 23217590 PMC3523892

[29] Committee on Accelerating Progress in Obesity Prevention; Food and Nutrition Board; Institute of Medicine. Accelerating Progress in Obesity Prevention: Solving the Weight of the Nation. In: Glickman D, Parker L, Sim LJ, Del Valle Cook H, Miller EA, editors. Washington, DC: National Academies Press (2012). p. 1–462. 24830053

[30] Bleich SN Segal J Wu Y Wilson R Wang Y. Systematic review of community-based childhood obesity prevention studies. Pediatrics. (2013) 132:e201–10. 10.1542/peds.2013-0886 23753099 PMC3691541

[31] Kumanyika S Grier S. Targeting interventions for ethnic minority and low-income populations. Fut Child. (2006) 16:187–207. 10.1353/foc.2006.0005 16532664

[32] Dietz WH. We need a new approach to prevent obesity in low-income minority populations. Pediatrics. (2019) 143:e20190839. 10.1542/peds.2019-0839 31126970

[33] Roberto CA Swinburn B Hawkes C Huang TT Costa SA Ashe M. Patchy progress on obesity prevention: emerging examples, entrenched barriers, and new thinking. Lancet. (2015) 385:2400–9. 10.1016/S0140-6736(14)61744-X 25703111

[34] Huang TT Cawley JH Ashe M Costa SA Frerichs LM Zwicker L. Mobilisation of public support for policy actions to prevent obesity. Lancet. (2015) 385:2422–31. 10.1016/S0140-6736(14)61743-8 25703113

[35] Islam NS Wyatt LC Ali SH Zanowiak JM Mohaimin S Goldfeld K. Integrating community health workers into community-based primary care practice settings to improve blood pressure control among South Asian immigrants in New York City: results from a randomized control trial. Circ Cardiovasc Qual Outcomes. (2023) 16:e009321. 10.1161/CIRCOUTCOMES.122.009321 36815464 PMC10033337

[36] Kumar V Kumar A Ghosh AK Samphel R Yadav R Yeung D. Enculturating science: Community-centric design of behavior change interactions for accelerating health impact. Semin Perinatol. (2015) 39:393–415. 10.1053/j.semperi.2015.06.010 26215599

[37] Swierad E Huang TT Ballard E Flórez K Li S. Developing a socioculturally nuanced systems model of childhood obesity in Manhattan's Chinese American community via group model building. J Obes. (2020) 2020:4819143. 10.1155/2020/4819143 33628493 PMC7895604

[38] Chambers DA. Advancing adaptation of evidence-based interventions through implementation science: progress and opportunities. Front Health Serv. (2023) 3:1204138. 10.3389/frhs.2023.1204138 37342795 PMC10277471

[39] Prochaska JO Velicer WF. The transtheoretical model of health behavior change. Am J Health Promot. (1997) 12:38–48. 10.4278/0890-1171-12.1.38 10170434

[40] de Freitas PP de Menezes MC Dos Santos LC Pimenta AM Ferreira AVM Lopes ACS. The transtheoretical model is an effective weight management intervention: a randomized controlled trial. BMC Public Health. (2020) 20:652. 10.1186/s12889-020-08796-1 32393214 PMC7216547

[41] Mastellos N Gunn LH Felix LM Car J Majeed A. Transtheoretical model stages of change for dietary and physical exercise modification in weight loss management for overweight and obese adults. Cochrane Database Syst Rev. (2014) 2014:Cd008066. 10.1002/14651858.CD008066.pub3 24500864 PMC10088065

[42] Hayotte M Nègre V Gray L Sadoul JL d'Arripe-Longueville F. The transtheoretical model (TTM) to gain insight into young women's long-term physical activity after bariatric surgery: a qualitative study. Obesity surgery. (2020) 30:595–602. 10.1007/s11695-019-04220-9 31654341

[43] Crabtree VM Moore JB Jacks DE Cerrito P Topp RV A. transtheoretical, case management approach to the treatment of pediatric obesity. J Prim Care Community Health. (2010) 1:4–7. 10.1177/2150131909357069 23804060

[44] Karasu SR. Psychotherapy-lite: obesity and the role of the mental health practitioner. Am J Psychother. (2013) 67:3–22. 10.1176/appi.psychotherapy.2013.67.1.3 23682511

[45] Livia B Elisa R Claudia R Roberto P Cristina A Emilia ST. Stage of change and motivation to a healthier lifestyle before and after an intensive lifestyle intervention. J Obes. (2016) 2016:6421265. 10.1155/2016/6421265 27239339 PMC4864539

[46] Puhl RM Lessard LM. Weight stigma in youth: prevalence, consequences, and considerations for clinical practice. Curr Obes Rep. (2020) 9:402–11. 10.1007/s13679-020-00408-8 33079337

[47] Tomiyama AJ. Stress and obesity. Annu Rev Psychol. (2019) 70:703–18. 10.1146/annurev-psych-010418-102936 29927688

[48] Adam TC Epel ES. Stress, eating and the reward system. Physiol Behav. (2007) 91:449–58. 10.1016/j.physbeh.2007.04.011 17543357

[49] Alhalel N Schueller SM O'Brien MJ. Association of changes in mental health with weight loss during intensive lifestyle intervention: does the timing matter? Obes Sci Pract. (2018) 4:153–8. 10.1002/osp4.157 29670753 PMC5893461

[50] Moroshko I Brennan L O'Brien P. Predictors of dropout in weight loss interventions: a systematic review of the literature. Obes Rev. (2011) 12:912–34. 10.1111/j.1467-789X.2011.00915.x 21815990

[51] Grootens-Wiegers P van den Eynde E Halberstadt J Seidell JC Dedding C. The “Stages Towards Completion Model”: what helps and hinders children with overweight or obesity and their parents to be guided towards, adhere to and complete a group lifestyle intervention. Int J Qual Stud Health Well-being. (2020) 15:1735093. 10.1080/17482631.2020.1735093 32148191 PMC7144242

[52] Byrd-Bredbenner C Eck KM. Relationships among executive function, cognitive load, and weight-related behaviors in university students. Am J Health Behav. (2020) 44:691–703. 10.5993/AJHB.44.5.12 33121586

[53] Sisakhti M Sachdev PS. Batouli SAH. The effect of cognitive load on the retrieval of long-term memory: an fMRI study. Front Hum Neurosci. (2021) 15:700146. 10.3389/fnhum.2021.700146 34720904 PMC8548369

[54] Howard ZL Evans NJ Innes RJ Brown SD Eidels A. How is multi-tasking different from increased difficulty? Psychon Bull Rev. (2020) 27:937–51. 10.3758/s13423-020-01741-8 32440999

[55] Siregar NR. Explicit instruction and executive functioning capacity: a new direction in cognitive load theory. J Educ. (2023) 203:451–8. 10.1177/00220574211033256

[56] Geiker NRW Astrup A Hjorth MF Sjödin A Pijls L Markus CR. Does stress influence sleep patterns, food intake, weight gain, abdominal obesity and weight loss interventions and vice versa? Obes Rev. (2018) 19:81–97. 10.1111/obr.12603 28849612

[57] Cox TL Krukowski R Love SJ Eddings K DiCarlo M Chang JY. Stress management-augmented behavioral weight loss intervention for African American women: a pilot, randomized controlled trial. Health Educ Behav. (2013) 40:78–87. 10.1177/1090198112439411 22505570

[58] Christaki E Kokkinos A Costarelli V Alexopoulos EC Chrousos GP Darviri C. Stress management can facilitate weight loss in Greek overweight and obese women: a pilot study. J Hum Nutr Diet. (2013) 26 (Suppl. 1):132–9. 10.1111/jhn.12086 23627835

[59] Huang TT Drewnosksi A Kumanyika S Glass TA. A systems-oriented multilevel framework for addressing obesity in the 21st century. Prevent. Chron. Dis. (2009) 6:A82. Available online at: https://www.ncbi.nlm.nih.gov/pmc/articles/PMC2722412/pdf/PCD63A82.pdf 19527584 PMC2722412

[60] Ash T Agaronov A Young T Aftosmes-Tobio A Davison KK. Family-based childhood obesity prevention interventions: a systematic review and quantitative content analysis. Int J Behav Nutr Phys Act. (2017) 14:113. 10.1186/s12966-017-0571-2 28836983 PMC5571569

[61] Collins LM Murphy SA Strecher V. The multiphase optimization strategy (MOST) and the sequential multiple assignment randomized trial (SMART): new methods for more potent eHealth interventions. Am J Prev Med. (2007) 32:S112–8. 10.1016/j.amepre.2007.01.022 17466815 PMC2062525

[62] Ventura AK Birch LL. Does parenting affect children's eating and weight status? Int J Behav Nutr Phys Act. (2008) 5:15. 10.1186/1479-5868-5-15 18346282 PMC2276506

[63] Kuhl ES Clifford LM Stark LJ. Obesity in preschoolers: behavioral correlates and directions for treatment. Obesity. (2012) 20:3–29. 10.1038/oby.2011.201 21760634

[64] Kugler KC Balantekin KN Birch LL Savage JS. Application of the multiphase optimization strategy to a pilot study: an empirical example targeting obesity among children of low-income mothers. BMC Public Health. (2016) 16:1181. 10.1186/s12889-016-3850-y 27876027 PMC5120514

[65] Alrige M Bitar H Meccawy M. Promoting precautionary behavior during the COVID-19 pandemic: development and validation of a behavior-change messaging campaign. J Infect Public Health. (2021) 14:1727–32. 10.1016/j.jiph.2021.09.026 34710783 PMC8491996

[66] Sittig S Wang J Iyengar S Myneni S Franklin A. Incorporating behavioral trigger messages into a mobile health app for chronic disease management: randomized clinical feasibility trial in diabetes. JMIR mHealth and uHealth. (2020) 8:e15927. 10.2196/15927 32175908 PMC7105932

[67] Sporrel K Wang S Ettema DDF Nibbeling N Krose BJA Deutekom M. Just-in-time prompts for running, walking, and performing strength exercises in the built environment: 4-week randomized feasibility study. JMIR formative research. (2022) 6:e35268. 10.2196/35268 35916693 PMC9379785

[68] Agha S Tollefson D Paul S Green D Babigumira JB. Use of the Fogg Behavior Model to Assess the Impact of a Social Marketing Campaign on Condom Use in Pakistan. J Health Commun. (2019) 24:284–92. 10.1080/10810730.2019.1597952 30945612

[69] Fogg B. A behavior model for persuasive design. In: Persuasive ‘09: Proceedings of the 4th International Conference on Persuasive Technology. (2009). p. 1–7. 10.1145/1541948.1541999

[70] Winett RA Davy BM Savla J Marinik EL Winett SG Baugh ME. Using response variation to develop more effective, personalized behavioral medicine?: evidence from the Resist Diabetes study. Transl Behav Med. (2014) 4:333–8. 10.1007/s13142-014-0263-2 25264472 PMC4167893

[71] Sherwood NE Butryn ML Forman EM Almirall D Seburg EM Lauren Crain A. The BestFIT trial: A SMART approach to developing individualized weight loss treatments. Contemp Clin Trials. (2016) 47:209–16. 10.1016/j.cct.2016.01.011 26825020 PMC4941634

[72] Unick JL Pellegrini CA Demos KE Dorfman L. Initial weight loss response as an indicator for providing early rescue efforts to improve long-term treatment outcomes. Curr Diab Rep. (2017) 17:69. 10.1007/s11892-017-0904-1 28726155 PMC5789799

[73] Hovmand PS Ford DN. Sequence and timing of three community interventions to domestic violence. Am J Community Psychol. (2009) 44:261–72. 10.1007/s10464-009-9264-6 19838793

[74] Guynn I Simon J Anderson S Klaman SL Mullenix A Cilenti D. Tools for supporting the MCH workforce in addressing complex challenges: a scoping review of system dynamics modeling in maternal and child health. Matern Child Health J. (2022) 26:176–203. 10.1007/s10995-022-03376-8 35188621 PMC9482604

[75] Naumann RB Austin AE Sheble L Lich KH. System dynamics applications to injury and violence prevention: a systematic review. Curr Epidemiol Rep. (2019) 6:248–62. 10.1007/s40471-019-00200-w 31911889 PMC6945820

[76] MacDermid JC Law M Buckley N Haynes RB. “Push” versus “Pull” for mobilizing pain evidence into practice across different health professions: a protocol for a randomized trial. Implement Sci. (2012) 7:115. 10.1186/1748-5908-7-115 23176444 PMC3520813

[77] Wallace AS Wang CY Flake N Bristol AA Altizer R. Feasibility and usefulness of the going home toolkit, an mhealth app, during hospital discharge: patient and clinician perspectives. Inform Health Soc Care. (2023) 48:1–12. 10.1080/17538157.2022.2043330 35234556

[78] Al-Badri M Kilroy CL Shahar JI Tomah S Gardner H Sin M. In-person and virtual multidisciplinary intensive lifestyle interventions are equally effective in patients with type 2 diabetes and obesity. Ther Adv Endocrinol Metab. (2022) 13:20420188221093220. 10.1177/20420188221093220 35464878 PMC9019312

[79] Lacagnina S Tips J Pauly K Cara K Karlsen M. Lifestyle medicine shared medical appointments. Am J Lifestyle Med. (2021) 15:23–7. 10.1177/1559827620943819 33456418 PMC7781059

[80] Branch A. American College of Lifestyle Medicine Releases Statement Calling for Compassionate, Evidence-Based Lifestyle Intervention as First Treatment for Overweight and Obesity. Chesterfield, MO: American College of Lifestyle Medicine (2023).

[81] Leung MM Tripicchio G Agaronov A Hou N. Manga comic influences snack selection in Black and Hispanic New York City youth. J Nutr Educ Behav. (2014) 46:142–7. 10.1016/j.jneb.2013.11.004 24433817

[82] Okorodudu DE Bosworth HB Corsino L. Innovative interventions to promote behavioral change in overweight or obese individuals: a review of the literature. Ann Med. (2015) 47:179–85. 10.3109/07853890.2014.931102 25011006 PMC4398644

[83] Turner-McGrievy GM Campbell MK Tate DF Truesdale KP Bowling JM Crosby L. Pounds off digitally study: a randomized podcasting weight-loss intervention. Am J Prev Med. (2009) 37:263–9. 10.1016/j.amepre.2009.06.010 19765496 PMC2892173

[84] Islam H Gibala MJ Little JP. Exercise snacks: a novel strategy to improve cardiometabolic health. Exerc Sport Sci Rev. (2022) 50:31–7. 10.1249/JES.0000000000000275 34669625

[85] Carey L. Taking BMI off the table. N Z Med J. (2019) 132:77–80. 31778375

[86] Burkhauser RV Cawley J. Beyond BMI: the value of more accurate measures of fatness and obesity in social science research. J Health Econ. (2008) 27:519–29. 10.1016/j.jhealeco.2007.05.005 18166236

[87] Sandborg J Söderström E Henriksson P Bendtsen M Henström M Leppänen MH. Effectiveness of a smartphone app to promote healthy weight gain, diet, and physical activity during pregnancy (HealthyMoms): randomized controlled trial. JMIR mHealth and uHealth. (2021) 9:e26091. 10.2196/26091 33704075 PMC7995071

[88] Nollen NL Mayo MS Carlson SE Rapoff MA Goggin KJ Ellerbeck EF. Mobile technology for obesity prevention: a randomized pilot study in racial- and ethnic-minority girls. Am J Prev Med. (2014) 46:404–8. 10.1016/j.amepre.2013.12.011 24650843 PMC3962588

[89] Bonvicini L Pingani I Venturelli F Patrignani N Bassi MC Broccoli S. Effectiveness of mobile health interventions targeting parents to prevent and treat childhood obesity: systematic review. Prevent Med Rep. (2022) 29:101940. 10.1016/j.pmedr.2022.101940 36161123 PMC9501985

[90] Ang SM Chen J Liew JH Johal J Dan YY Allman-Farinelli M. Efficacy of interventions that incorporate mobile apps in facilitating weight loss and health behavior change in the Asian population: systematic review and meta-analysis. J Med Internet Res. (2021) 23:e28185. 10.2196/28185 34783674 PMC8663646

[91] Eisenhauer CM Brito F Kupzyk K Yoder A Almeida F Beller RJ. Mobile health assisted self-monitoring is acceptable for supporting weight loss in rural men: a pragmatic randomized controlled feasibility trial. BMC Public Health. (2021) 21:1568. 10.1186/s12889-021-11618-7 34407782 PMC8375071

[92] Ogden J Maxwell H Wong A. Development and feasibility study of an app (Ladle) for weight loss and behaviour change. PeerJ. (2019) 7:e6907. 10.7717/peerj.6907 31143540 PMC6525583

[93] Nikolaou CK Tay Z Leu J Rebello SA Te Morenga L Van Dam RM. Young people's attitudes and motivations toward social media and mobile apps for weight control: mixed methods study. JMIR mHealth and uHealth. (2019) 7:e11205. 10.2196/11205 31603431 PMC6913715

[94] University of Washington. What is Implementation Science? Wasington, DC: University of Washington. Available online at: https://impsciuw.org/implementation-science/learn/implementation-science-overview/ (accessed October 25, 2022).

[95] University of Washington. Theories, Models, and Frameworks. Available online at: https://impsciuw.org/implementation-science/research/frameworks/ (accessed January 4, 2023).

[96] Pinto RM Park S Miles R Ong PN. Community engagement in dissemination and implementation models: a narrative review. Implement Res Pract. (2021) 2:2633489520985305. 10.1177/2633489520985305 37089998 PMC9978697

[97] Peek ST Wouters EJ Luijkx KG Vrijhoef HJ. What it takes to successfully implement technology for aging in place: focus groups with stakeholders. J Med Internet Res. (2016) 18:e98. 10.2196/jmir.5253 27143097 PMC4904824

[98] Norman DA Draper SW. User Centered System Design; New Perspectives on Human-Computer Interaction. Mahwah, NJ: L Erlbaum Associates Inc.(1986).

[99] Haines ER Dopp A Lyon AR Witteman HO Bender M Vaisson G. Harmonizing evidence-based practice, implementation context, and implementation strategies with user-centered design: a case example in young adult cancer care. Implement Sci Commun. (2021) 2:45. 10.1186/s43058-021-00147-4 33902748 PMC8077816

